# Voluntary Medical Male Circumcision: Translating Research into the Rapid Expansion of Services in Kenya, 2008–2011

**DOI:** 10.1371/journal.pmed.1001130

**Published:** 2011-11-29

**Authors:** Zebedee Mwandi, Anne Murphy, Jason Reed, Kipruto Chesang, Emmanuel Njeuhmeli, Kawango Agot, Emma Llewellyn, Charles Kirui, Kennedy Serrem, Isaac Abuya, Mores Loolpapit, Regina Mbayaki, Ndungu Kiriro, Peter Cherutich, Nicholas Muraguri, John Motoku, Jack Kioko, Nancy Knight, Naomi Bock

**Affiliations:** 1 Division of Global HIV/AIDS, United States Centers for Disease Control and Prevention, Nairobi, Kenya; 2 United States Agency for International Development, Nairobi, Kenya; 3 Division of Global HIV/AIDS, Centers for Disease Control and Prevention, Atlanta, Georgia, United States of America; 4 United States Agency for International Development, Washington, District of Columbia, United States of America; 5 Impact Research and Development Organization, Kisumu, Kenya; 6 Nyanza Reproductive Health Society, Kisumu, Kenya; 7 Kenya Medical Research Institute—Family AIDS and Care Education Services, Nairobi, Kenya; 8 Catholic Medical Missions Board, Nairobi, Kenya; 9 C-Change Communication for Change, Nairobi, Kenya; 10 Family Health International, Nairobi, Kenya; 11 Engender Health (APHIA II Nyanza), Kisumu, Kenya; 12 Population Services International, Nairobi, Kenya; 13 Kenya National AIDS and STD Control Programme, Nairobi, Kenya; 14 Eastern Deanery AIDS Response Program, Nairobi, Kenya; 15 Ministry of Public Health and Sanitation, Kisumu, Kenya; Centers for Disease Control and Prevention, United States of America

## Abstract

Since the World Health Organization and the Joint United Nations Programme on HIV/AIDS recommended implementation of medical male circumcision (MC) as part of HIV prevention in areas with low MC and high HIV prevalence rates in 2007, the government of Kenya has developed a strategy to circumcise 80% of uncircumcised men within five years. To facilitate the quick translation of research to practice, a national MC task force was formed in 2007, a medical MC policy was implemented in early 2008, and Nyanza Province, the region with the highest HIV burden and low rates of circumcision, was prioritized for services under the direction of a provincial voluntary medical male circumcision (VMMC) task force. The government's early and continuous engagement with community leaders/elders, politicians, youth, and women's groups has led to the rapid endorsement and acceptance of VMMC. In addition, several innovative approaches have helped to optimize VMMC scale-up. Since October 2008, the Kenyan VMMC program has circumcised approximately 290,000 men, mainly in Nyanza Province, an accomplishment made possible through a combination of governmental leadership, a documented implementation strategy, and the adoption of appropriate and innovative approaches. Kenya's success provides a model for others planning VMMC scale-up programs.

## Abstract 2

Zebedee Mwandi and colleagues discuss Kenya's scale-up of voluntary medical male circumcision services, highlighting government leadership, a clear implementation strategy, and program flexibility and innovation as keys to Kenya's success.

**Summary Points**

- Kenya's male circumcision for HIV prevention policy prioritizes Nyanza Province, the region with the highest HIV burden and low circumcision rates, for scale-up of voluntary medical male circumcision (VMMC) services.
- Since the policy's implementation in October 2008, approximately 290,000 adult males have been circumcised in Kenya, most of them in Nyanza Province.
- Government leadership and a documented implementation strategy have been key factors in Kenya's rapid scale-up of VMMC.
- Another key factor has been program flexibility: the introduction of innovative approaches, including task shifting, short intensive service campaigns, and, most recently, diathermy for hemostasis, have all helped the program respond to challenges.
- Kenya's successful approach to VMMC scale-up provides a model that other countries can adapt to their own circumstances.

## Introduction

In the past two decades, observational studies have provided increasing evidence that male circumcision (MC) has an HIV prevention effect [1]. Moreover, three randomized controlled trials have reported that medical circumcision of men reduces HIV acquisition from infected female partners by approximately 60% [2]–[4]. This evidence led the World Health Organization (WHO) and the Joint United Nations Programme on HIV/AIDS (UNAIDS) to issue recommendations in 2007 that countries should include medical MC as part of HIV prevention interventions and that implementation should be prioritized in areas with low MC and high HIV prevalence rates [5]. WHO and UNAIDS identified 13 priority countries for scale-up of medical MC. The United States President's Emergency Plan for AIDS Relief (PEPFAR) is supporting activities to implement medical MC in these 13 countries plus the Gambella National Regional State in Ethiopia (Table 1).

**Table 1:** Target number of HIV-negative males needed to be medically circumcised by 2015 to reach 80% coverage, and approximate proportion of those reached with medical MC services through late 2011.

Country	Target Number of 15- to 49-Year-Old, HIV-Negative, Uncircumcised Males (Approximate)	Approximate Percentage Circumcised since 2007 WHO Recommendations (Rounded)
Botswana	345,000	5
Ethiopa: Gambella National Regional State	40,000	15
Kenya: Nyanza Province	380,000^a^/426,000^b^	55^a^/50^b^
Lesotho	377,000	<5
Malawi	2,102,000	<5
Moazmbique	1,059,000	<5
Namibia	330,000	<5
Rwanda	1,746,000	<5
South Africa	4,333,000	<5
Swaziland	183,000	15
Tanzania	1,373,000	<5
Uganda	4,250,000	<5
Zambia	1,949,000	<5
Zimbabwe	1,913,000	<5

a Estimate and calculation based upon Decision Makers' Program Planning Tool [9].

b Estimate and calculation based upon the Kenya national strategy for VMMC [19].

Nyanza Province in Kenya is one of the regions in sub-Saharan Africa prioritized by WHO and UNAIDS for implementation of medical MC (Table 1). Although more than 80% of men in Kenya are circumcised [6], MC coverage varies culturally and geographically. Nyanza Province, which is largely Luo, has the lowest MC coverage (48%) and the highest prevalence of HIV (14.9%) in Kenya [6],[7]. Given these data and the WHO recommendations, the government of Kenya, through the Ministry of Health, recognizes medical MC as an additional and important strategy for the prevention of heterosexually acquired HIV infection in men and has developed a national strategy that aims to circumcise 80% of uncircumcised HIV-negative men aged 15–49 years (approximately 860,000 men throughout the country, 426,000 in Nyanza Province alone) between 2009 and 2013 [8]. Modeling studies conducted by the United States President's Emergency Plan for AIDS Relief and UNAIDS estimate that a scale-up strategy of 80% medical MC coverage in five years in Nyanza Province could avert an estimated 45,000 new HIV infections over 15 years [9].

Kenya has already made good progress towards meeting its MC target (Table 1). By contrast, most other MC scale-up priority countries in sub-Saharan Africa have made less progress towards meeting their MC targets [10] (Table 1). In this case study, we describe the approach that Kenya has taken to translating research into policy and program, and identify three key factors—government leadership, program flexibility, and a documented implementation strategy—that have facilitated Kenya's early success in the scale-up of medical MC. We also discuss the lessons learned and the challenges that still need to be overcome before Kenya can reach its MC target.

## Government Leadership

The Kenyan Ministry of Health and the National AIDS and STI Control Programme (NASCOP) began providing leadership on medical MC for HIV prevention before the conclusion of the randomized controlled trials mentioned above and before WHO issued its recommendations in 2007 [5]. At a consultative meeting held in Nairobi in September 2006, researchers, policy makers, donors, and other stakeholders discussed how Kenya should respond to the results of the Kisumu and Rakai randomized controlled trials, whether positive or negative. In December 2006, when these studies were stopped early because of MC's overwhelming efficacy in reducing HIV transmission risk, Kenya's Director of Medical Services issued a statement calling for the establishment of a national MC task force to advise the government on how to proceed. The “National Guidance for Voluntary Male Circumcision in Kenya”—the first national MC policy in sub-Saharan Africa—was drafted by this task force, approved in December 2007, and published in January 2008 [11]. To complement the work of the Kenya national MC task force, a Nyanza Province MC task force and district coordinating bodies were also established in early 2007, and assessments of health facilities in Nyanza Province were conducted to determine the province's preparedness to provide VMMC services. Gaps were identified and remedied with support from international donors.

While work on the national policy document was proceeding, the Kenyan government took steps to engage the Luo Council of Elders in Nyanza Province in the scale-up of medical MC. To gain the support of these protectors of Luo culture for medical MC scale-up, the government needed to explain to them why medical MC would be recommended for HIV prevention and how medical MC was biologically protective against the HIV virus. In addition, the government needed to improve its understanding of the council's potential concerns. Repeated discussions satisfied the Luo Council of Elders that MC for HIV prevention would be voluntary and provided for medical and not cultural reasons. As a result, the term “voluntary medical male circumcision” (VMMC) was officially adopted in Kenya, instead of just “male circumcision.”

The day before the official launch of VMMC services in October 2008, three community-based stakeholders' meetings were held with cultural leaders, government ministers (including the prime minister of Kenya, Raila Odinga, and the minister of health), local politicians, youth, religious and women's groups, and health professionals. In addition, several members of parliament and cabinet ministers publicly disclosed that they were circumcised, as a show of support for Kenya's VMMC programs for HIV prevention.

## Program Flexibility

### Service Delivery Approaches

Service delivery providers have implemented VMMC services in Nyanza Province using a variety of site and staffing models. Permanent sites in larger health care facilities staffed with existing local health care personnel, outreach services that temporarily deploy health care teams to smaller health facilities, and mobile services that temporarily deploy health care teams to non-health-care facilities such as churches, schools, or tents have all been used. These sites are both public and private, as are staff, and staffing models have frequently included government and non-government staff working side by side. Drawing upon a variety of public and private site and staffing models, instead of restricting services to a particular model, has produced a program that is flexible and adaptable.

Twelve months after the VMMC program began, only 46,000 boys and men had been circumcised, and officials recognized that they would not reach the program's target. Consequently, NASCOP and the Ministry of Health introduced a Rapid Results Initiative (RRI) approach as an additional service delivery model. The RRI is a public health service delivery strategy that focuses on short-term, results-oriented activities designed to reach a high number of individuals quickly; RRIs have been previously undertaken in Kenya for HIV testing and counseling campaigns and immunization campaigns [12].

The first VMMC RRI, which ran in November and December 2009 to coincide with school holidays, aimed to circumcise 30,000 adolescent and adult males in 11 districts in Nyanza Province in 30 working days [12]. Tents were used extensively during the campaign to provide services in areas where more permanent infrastructure was lacking. As with all VMMC services for HIV prevention, in addition to providing the MC surgical procedure, the RRI offered each client a comprehensive HIV prevention package that included HIV testing and counseling, screening and treatment for sexually transmitted infections, and promotion and provision of condoms. A second VMMC RRI, which ran in November and December 2010, aimed to circumcise 46,000 men.

### Human Resources

In many countries in sub-Saharan Africa, only physicians (medical officers) may perform medical MC. In task shifting, a trained individual from a less educated health care cadre (a non-physician) is permitted to perform a medical procedure. Right from the launch of the national VMMC policy, Kenya permitted clinical officers to perform VMMC in addition to medical officers, thus providing an expanded pool of health care providers who could perform VMMC. However, health facilities were understaffed with clinical officers and medical officers, and in 2009, the director of medical services, on the recommendation of the Kenyan national MC task force, supported further task shifting to allow nurses to perform VMMC, thereby ensuring that there would be sufficient human resources available to achieve the goals of the VMMC program. Task shifting of VMMC to nurses likely also helps to preserve the pool of clinical and medical officers able to meet the other demands of the Kenyan health care system, but research is needed to confirm this possibility.

### Clinical Techniques

Because trained personnel are in short supply—even with task shifting—making the most efficient use of health care providers' time is important to maximize productivity. The WHO guidance “Considerations for Implementing Models for Optimizing the Volume and Efficiency of Male Circumcision Services for HIV Prevention” summarizes options for improving service productivity, without compromising safety and quality [13], including the time-saving advantages of diathermy for hemostasis. Subsequent to the release and wide adoption of the service delivery models proposed by WHO, the Kenyan VMMC program has begun incorporating the use of diathermy to improve program efficiency and productivity.

## Implementation Strategy

The Kenyan government operationalized its national policy in a written implementation strategy that is part of the Kenya National AIDS Strategic Plan [8]. The implementation strategy provides guidance on the quality of service delivery, delivery of services with full human rights' considerations, correct dissemination of information within the context of broader HIV prevention interventions, demand creation, and monitoring and evaluation. It employs a three-phased approach, consisting of short-, medium-, and long-term objectives, and outlines annual targets and milestones for each region, through 2013. To date, implementation and service delivery have been concentrated in Nyanza Province.

From the outset, as part of its implementation strategy, the program has focused on strategies for social mobilization, advocacy, and health communication. To achieve and sustain social mobilization, journalists have been educated about the science underlying the use of VMMC for HIV prevention to ensure accurate reporting of the national strategy and program and to help create a positive public perception of VMMC. Although the initial national-level advocacy yielded tremendous gains, program partners have also engaged in intense consultation with gatekeepers at the community level. Finally, program partners have sensitized health workers to the role of VMMC within the context of HIV prevention programming.

## Achievements

**Figure 1:**
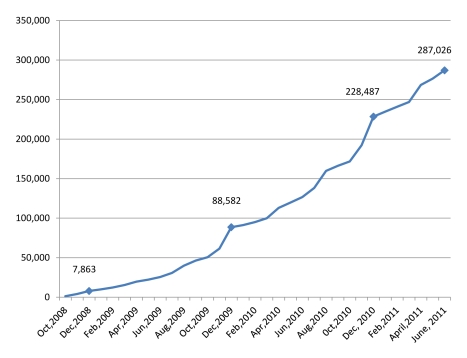
Cumulative circumcisions done in Kenya, 2008–2011.

**Figure 2:**
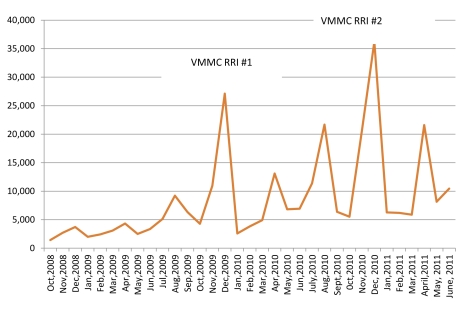
Monthly circumcisions done in Kenya, 2008–2011.

Since October 2008, the Kenyan VMMC program has circumcised approximately 290,000 men, mainly in Nyanza Province (Figures 1 and 2), and more than 700 providers of various cadres have been trained to provide VMMC services. Although the 2009 and 2010 RRIs, which completed about 36,000 and 50,000 VMMCs, respectively (personal communication, A. Ochieng, NASCOP), boosted the overall number of men circumcised in Kenya, monthly performances outside these periods have increased from as low as 3,000 VMMCs in the first ten months (October 2008–July 2009) to an average of about 6,000 VMMCs in recent months (May 2010–June 2011). Improvements in service efficiency, dedication of full-time space and staff, increased demand for services, and greater availability of outreach/mobile services have all likely contributed to higher overall service numbers outside the RRIs.

The quality of service delivery has also increased over the life of the project. For example, uptake of HIV testing among VMMC clients at Nyanza Reproductive Health Society—one the largest providers of VMMC services in Kenya—has increased since the beginning of the program, from 31% in 2008–2009 to more than 83% presently, largely because of a shift to a provider-initiated HIV testing approach from opt-in HIV testing (personal communication, A. Ochieng, NASCOP).

In addition, a routine clinical record and reporting system has been adopted by all service providers, with a standard set of intra-operative and postoperative adverse event definitions, based on WHO guidance [14]. Adverse event occurrences, along with other service statistics, are now aggregated and reported through health management and information systems to the Ministry of Health for review by the national and provincial MC task forces. Overall, moderate and severe adverse event rates have remained at or below 3% since 2009 (personal communication, A. Ochieng, NASCOP).

Finally, the proportion of men aged 15 years or older undergoing VMMC has increased over time from approximately 55% in the 2009 VMMC RRI to 84% in the 2010 RRI (personal communication, A. Ochieng, NASCOP), an encouraging result, given that preferential targeting of VMMC to males who are now or soon will be sexually active is needed to accelerate the prevention impact of VMMC programs.

## Lessons Learned

The experiences of Kenya's VMMC program suggest that early engagement of traditional leaders from non-circumcising communities can benefit national policy and implementation strategy development processes. They also suggest that flexible expansion of task shifting to allow nurses to perform medical MC can lessen human resource constraints without compromising safety [15]. Moreover, they indicate that retooling the implementation strategy to include mobile and outreach services and RRIs can effectively increase the uptake of VMMC. Finally, anecdotal best practice from the field suggests that using diathermy for hemostasis can improve efficiency [13]; service providers are now beginning to train health care workers on the use of diathermy in an effort to further increase productivity.

## Challenges

Despite the general success of the Kenyan VMMC program, several challenges remain. First, more must be done to overcome barriers among older men to go for VMMC services. These barriers include hesitations about taking time off work after surgery and particular concerns about abstaining from sex during wound healing among married men [16]. Presently, the national and provincial MC task forces are piloting new approaches to recruit older men to services, such as utilizing older circumcised clients as community mobilizers and providing incentives for these community mobilizers when older men present to VMMC facilities for information.

As VMMC is considered primarily a man's issue, involving women in VMMC programs can be a challenge. However, studies have shown that women play a large role in men's decision to be circumcised [17]. Kenya's VMMC program has made efforts to reach out to women by encouraging HIV testing and counseling of couples, by targeting women with gender-focused communication campaigns, and by urging men to involve their partners in the decision-making process. A comprehensive communication campaign has been implemented recently that addresses demand creation and women's roles in men's decisions about VMMC [18].

Kenya also needs to determine the best way to expand VMMC services to other regions, communities, and cultures, and to decide whether to integrate youth and neonatal medical MC into existing health services or to make them standalone services like the adolescent/adult VMMC program. Specifically, the sustainability of medical MC through the implementation of neonatal circumcision, which is not a common practice in Kenya, must be explored.

## Conclusion

The Kenyan VMMC experience has shown that with strong leadership from the government (the Kenyan government has assumed visible ownership of the VMMC program throughout its development and implementation and has focused stakeholders' attention on the number of HIV infections likely to be averted through VMMC scale-up), and with the enthusiastic participation of stakeholders, it is possible to initiate and expand VMMC in a short period of time. The Kenyan VMMC program—one of the first successful early translations of MC health research into implementation—provides a model that may help guide other countries in the region that are experiencing a slower scale-up of their VMMC programs. In particular, the engagement of traditional/community leaders, the establishment of national and local leadership bodies, and Kenya's willingness to consider multiple approaches to deal with implementation challenges hold important lessons for other countries. Finally, the experience of Kenya's VMMC program emphasizes the importance of having a comprehensive, timed, and actionable implementation strategy to which full-time staff from both the national government and agencies of foreign governments are dedicated.

## Abbreviations

MC: male circumcision
NASCOP: National AIDS and STI Control Programme
PEPFAR: United States President's Emergency Plan for AIDS Relief
RRI: Rapid Results Initiative
UNAIDS: Joint United Nations Programme on HIV/AIDS
VMMC: voluntary medical male circumcision
WHO: World Health Organization
